# Antifibrotics and mortality in idiopathic pulmonary fibrosis: external validity and avoidance of immortal time bias

**DOI:** 10.1186/s12931-024-02922-y

**Published:** 2024-07-31

**Authors:** Hironao Hozumi, Koichi Miyashita, Eiji Nakatani, Yusuke Inoue, Hideki Yasui, Yuzo Suzuki, Masato Karayama, Kazuki Furuhashi, Noriyuki Enomoto, Tomoyuki Fujisawa, Naoki Inui, Takafumi Suda

**Affiliations:** 1 Second Division, Department of Internal Medicine, Hamamatsu University School of Medicine, 1-20-1 Handayama Higashiku, Hamamatsu, 431-3192 Japan; 2 Research Support Center, Shizuoka General Hospital, 4-27-1 Kita Ando Aoi-ku, Shizuoka, 420-8527 Japan; 3 Department of Clinical Pharmacology and Therapeutics, Hamamatsu University School of Medicine, 1-20-1 Handayama Higashiku, Hamamatsu, 431-3192 Japan

**Keywords:** Idiopathic pulmonary fibrosis, Antifibrotics, Nintedanib, Pirfenidone, Mortality

## Abstract

**Background and objective:**

Pooled analyses of previous randomized controlled trials reported that antifibrotics improved survival in patients with idiopathic pulmonary fibrosis (IPF), but the results were only based on short-term outcome data from selected patients who met strict criteria. Observational studies/meta-analyses also suggested that antifibrotics improve survival, but these studies failed to control for immortal time bias that considerably exaggerates drug effects. Therefore, whether antifibrotics truly improve long-term survival in patients with IPF in the real world remains undetermined and requires external validity.

**Methods:**

We used data from the Japanese National Claims Database to estimate the intention-to-treat effect of antifibrotics on mortality. To address immortal time bias, we employed models treating antifibrotic initiation as a time-dependent covariate and target trial emulation (TTE), both incorporating new-user designs for antifibrotics and treating lung transplantation as a competing event.

**Results:**

Of 30,154 patients with IPF, 14,525 received antifibrotics. Multivariate Fine–Gray models with antifibrotic initiation as a time-dependent covariate revealed that compared with no treatment, nintedanib (adjusted hazard ratio [aHR], 0.85; 95% confidence interval [CI], 0.81–0.89) and pirfenidone (aHR, 0.89; 95% CI, 0.86–0.93) were associated with reduced mortality. The TTE model also replicated the associations of nintedanib (aHR, 0.69; 95% CI, 0.65–0.74) and pirfenidone (aHR, 0.81; 95% CI, 0.78–0.85) with reduced mortality. Subgroup analyses confirmed this association regardless of age, sex, and comorbidities, excluding certain subpopulations.

**Conclusions:**

The results of this large-scale real-world analysis support the generalizability of the association between antifibrotics and improved survival in various IPF populations.

**Supplementary Information:**

The online version contains supplementary material available at 10.1186/s12931-024-02922-y.

## Introduction

Idiopathic pulmonary fibrosis (IPF) is a progressive, fatal disease characterized by an irreversible decline in respiratory function [1]. Although randomized controlled trials (RCTs) have demonstrated that nintedanib and pirfenidone—the antifibrotics currently approved for IPF treatment worldwide—effectively slowed the IPF-related decline in respiratory function, [2–7] no RCT has proven that these drugs reduce the mortality risk. Pooled analyses of these RCTs suggest that antifibrotics have a per-protocol effect in reducing mortality [8, 9]. However, other meta-analyses have reported diverse results, including no reduction in mortality risk from treatment with either or both agents [10–13]. Additionally, the RCTs and their pooled analysis results are based on short-term outcome data (52–72 weeks) for selected patients who passed strict inclusion/exclusion criteria [14]. In real-world settings, patients who do not meet such strict criteria (i.e., those aged ≥ 80 years and those with multiple comorbidities) comprise substantial proportion of patients with IPF. Therefore, analyses based on long-term outcome data from a broad patient population are needed.

Several observational studies that have used intention-to-treat analyses have reported that antifibrotics positively affected survival [15–28]. However, these studies, including meta-analysis that integrated their results, [29] did not control for immortal time bias [30–32]. This is a significant bias that can considerably exaggerate the effects of a drug [31]. In this bias, immortal time refers to the period during which an outcome of interest (e.g., death) cannot occur in a study participant during the specified follow-up period. Immortal time can be created by setting a minimum duration of drug use when defining a treated patient group. The time between the start of observation and the start of treatment is also immortal time; however, excluding such time from the analysis of the treated group leads to immortal time bias [31]. In observational studies, both the misclassification and exclusion of potential periods of immortal time without adequately addressing it when defining the duration of drug exposure create a bias toward the positive effects of the drug on the treated group. Therefore, controlling for immortal time bias is an essential prerequisite to accurately estimate the effects of a drug. Additionally, the results of previous studies were mainly based on patients receiving medical care in hospitals specializing in interstitial lung disease (ILD). However, in the real world, some patients with IPF lack access to such specialized hospitals for various reasons, including eligibility and/or institutional/geographical/economic issues, and a significant number of patients receive medical care in hospitals that do not specialize in ILD. Therefore, whether antifibrotics truly improve long-term survival in various IPF populations has not been confirmed. In light of this perspective, it remains essential to generalize findings by establishing evidence with external validity for populations that are more representative of the real world. Owing to the progressive and fatal nature of IPF, performing an RCT in which antifibrotics are compared to a placebo to determine the effects of antifibrotics on mortality is not ethically feasible. Hence, in this study, data from a large population of IPF patients drawn from the Japanese National Claims Database (NDB) were analyzed. The primary objectives of this study were to analyze these data and determine the intention-to treat effects of antifibrotic therapy on mortality in patients with IPF using methods avoiding immortal time bias.

## Methods

### Patient data

**Fig. 1:**
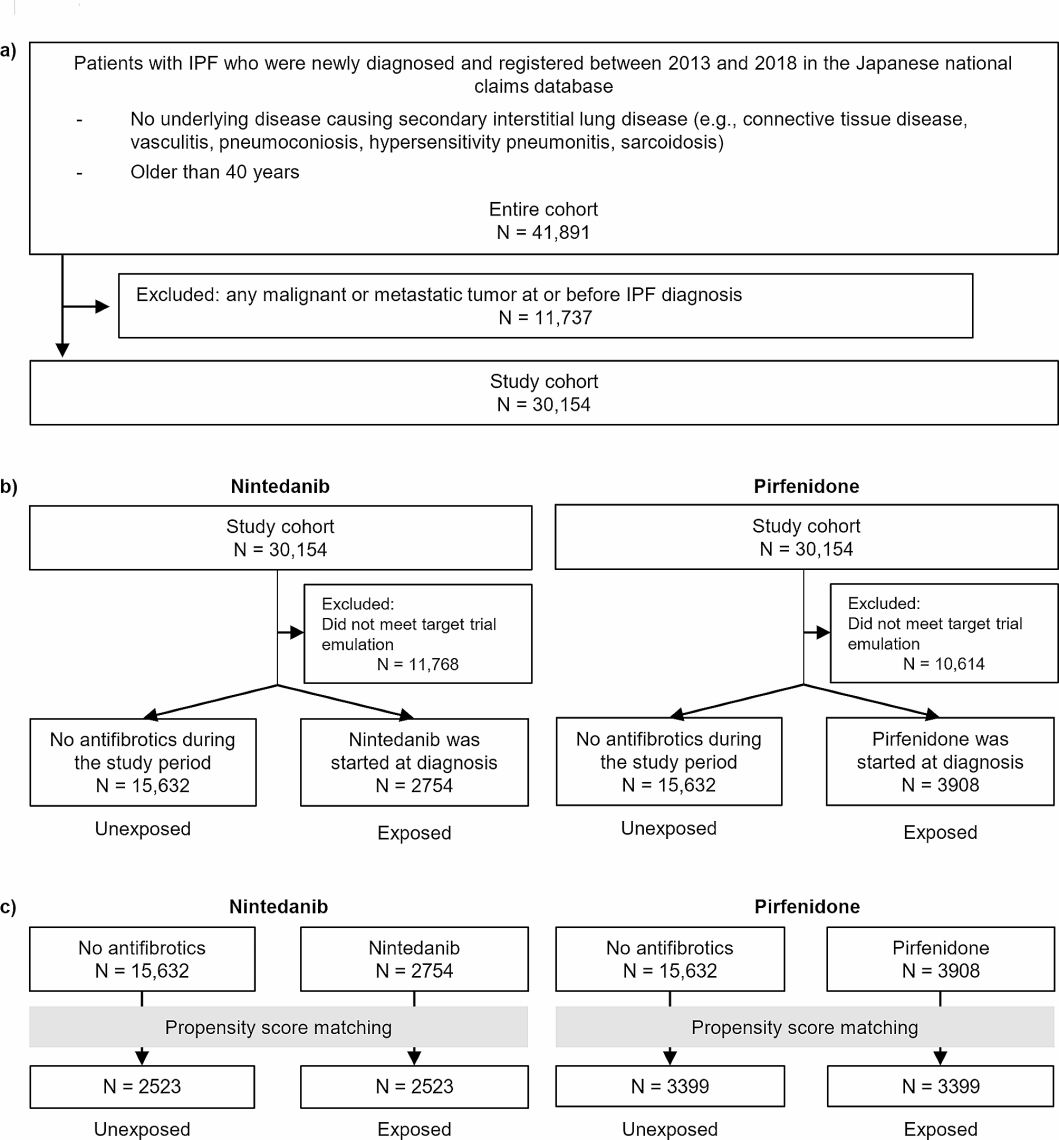
Flow diagram presenting the processes used for enrolling patients, grouping for target trial emulation, and propensity score matching. (**A**) Flow diagram of patient enrollment. (**B**) Flow diagram of grouping patients with idiopathic pulmonary fibrosis for target trial emulation with a new-user design for antifibrotics. Patients who had not used antifibrotics between the date of idiopathic pulmonary fibrosis (IPF) diagnosis and the date of censoring or death were categorized as unexposed patients, whereas those who started using antifibrotics on the date of IPF diagnosis were categorized as antifibrotic-exposed patients. The intention-to-treat analysis included the unexposed patients and all patients who were assigned to first-line antifibrotic treatment (nintedanib or pirfenidone). Multivariate Fine–Gray models were used in the intention-to-treat analyses of the effects of antifibrotics on mortality. (**C**) Flow diagram of propensity score matching. In the target trial emulation, propensity score-matched comparisons were made between antifibrotic-exposed and unexposed patients. Propensity scores were calculated using a logistic regression model adjusted for age, sex, cerebrovascular disease, dementia, acquired immunodeficiency syndrome/human immunodeficiency virus, myocardial infarction, renal disease, congestive heart failure, peripheral vascular disease, chronic pulmonary disease, peptic ulcer, liver disease, diabetes mellitus, hemiplegia or paraplegia, venous thromboembolic disease, pulmonary hypertension, long-term oxygen use, and corticosteroid use at baseline

Detailed patient information is provided in the Additional file: Appendix S1 in the Supporting Information. Figure 1A presents the flow diagram of patient enrollment. The NDB is one of the largest medical databases globally, containing medical data for over 126 million people and processing 1.9 billion claims annually, covering > 99% of Japanese medical claims. Given the widespread insurance coverage in Japan, data on nearly all patients diagnosed with IPF can be extracted from the NDB. Therefore, use of the NDB allows us to conduct studies with external validity that accurately represent the real world. We attempted to increase the specificity of IPF diagnosis by selecting patients using an ICD-10 code for IPF without a baseline or prior history of diseases that can cause secondary based on the algorithm by Raghu et al., with a slight modification [33]. We included 41,891 patients diagnosed with IPF and registered with the NDB between 2013 and 2018 (entire cohort). Of these, we excluded 11,737 patients who had an ICD-10 code for malignancy or metastatic tumor at or before the time of IPF diagnosis. Consequently, 30,154 patients were enrolled (study cohort). We extracted data on comorbid diseases used to calculate the Charlson comorbidity index, which is commonly used as a risk-adjusting variable (Additional file: Table S1), and data on venous thromboembolic disease and pulmonary hypertension, which have been reported as prognostic factors in IPF [34–36]. Patients were censored if they remained alive until December 31, 2019.

The guidelines of the Japanese Ministry of Health, Labour and Welfare (JMHLW) prohibit the publication of specific numbers of variables with fewer than 10 patients for anonymity reasons. Therefore, variables with fewer than 10 patients were denoted as < 10 or were not presented. Moreover, owing to extremely small number, those patients were excluded from the analysis for simultaneous treatment with nintedanib and pirfenidone except for those in Table 1.

**Table 1:** Clinical and demographic characteristics of study cohort

	*N* = 30,154
Baseline	
Median age category, years	75–79 ^a^
Men	21,908 (72.7)
Comorbidity category for the Charlson Comorbidity Index	
Cerebrovascular disease	7423 (24.0)
Dementia	1963 (5.6)
Acquired immunodeficiency syndrome/human immunodeficiency virus	12 (0.04)
Myocardial infarction	1788 (5.9)
Renal disease	2017 (6.7)
Congestive heart failure	9994 (33.1)
Peripheral vascular disease	5609 (18.6)
Chronic pulmonary disease	20,845 (69.1)
Peptic ulcer	9525 (31.6)
Liver disease	7832 (26.0)
Diabetes mellitus	13,822 (45.8)
Hemiplegia or paraplegia	413 (1.4)
Charlson comorbidity index	
0–2	15,297 (50.7)
3–4	10,057 (33.4)
≥ 5	4800 (15.9)
Venous thromboembolic disease	192 (0.6)
Pulmonary hypertension	931 (3.1)
Long-term oxygen use	4082 (13.5)
Corticosteroid use	7383 (24.5)
Observation period, months	21.6 (12.0–37.4)
Lung transplantation	22 (0.07)
Death	15,722 (52.1)
Antifibrotic treatment during the study period, yes	14,525 (48.2)
First-line antifibrotic therapy	
Nintedanib	6972
Pirfenidone	7542
Combined nintedanib and pirfenidone	11
Antifibrotic therapy duration, months	11.9 (3.0–23.5)
Antifibrotic discontinuation	6431 (44.3)

Data are presented as the median age category, median (interquartile range), or as number (%)

^a^ Details of the distribution of age categories are presented in Additional file: Figure S1 in the Supporting Information

### Controlling for immortal time bias

Treatment records for patients treated with nintedanib or pirfenidone were extracted, including the dates of treatment initiation and discontinuation. The methods used for avoiding immortal time bias were (1) models with drug initiation treated as a time-dependent covariate [31] and (2) a target trial emulation (TTE) framework, [37] both having new-user designs for antifibrotics. More details are provided in the Additional file: Appendix S2 in the Supporting Information.

The first model employed was Fine–Gray model with antifibrotic initiation as a time-dependent covariate to investigate the intention-to-treat effects of antifibrotics in the study cohort. All-cause mortality was treated as the event of interest, and lung transplantation was used as a competing event in this model. The analyses included all those patients who did not use antifibrotics between the dates of IPF diagnosis (i.e., baseline) and censoring or death (unexposed patients) and all those who were assigned for first-line antifibrotic treatment (nintedanib or pirfenidone) between the dates of IPF diagnosis and censoring or death.

The second model was a TTE framework that emulated a target trial. First, a hypothetical but pragmatic clinical trial was designed to answer the clinical question of interest (target trial specification), subsequently a TTE framework was designed to approximate the target trial using available observational data. Patients who met the specified criteria in the TTE framework were then enrolled and analyzed. As a target trial, this study emulated a trial comparing mortality between patients who were exposed and unexposed to antifibrotics. To avoid immortal time bias, we designated the date when patients met the eligibility criteria as time zero. Patients whose allocation to the treatment strategy coincided with this time zero date were identified, and follow-up was initiated on the same date (i.e., time zero; Additional file: Table S2). Hence, this model included “unexposed patients” and patients who initiated antifibrotic treatment on the date of IPF diagnosis (nintedanib- or pirfenidone-exposed patients; Fig. 1B). In this model, all-cause mortality was treated as the event of interest, and lung transplantation was served as a competing event. Multivariate Fine–Gray models were used in intention-to-treat analyses of the antifibrotic effects. The results were then validated using propensity score-matched comparisons between the antifibrotic-exposed and unexposed patients (Fig. 1C). Both analyses were adjusted for baseline confounders.

### Statistical analysis

Age data were expressed as age range categories (in increments of 5 years) in accordance with JMHLW guidelines. In all analyses, a *p*-value of < 0.05 was considered statistically significant. Standardized differences were also identified to assess differences in baseline variables between two groups. When the standardized difference was < 0.1, the inter-group variables were considered approximately equivalent even if the *p*-value was significant. More details are provided in the Additional file: Appendix S3.

## Results

### Study cohort characteristics

Among all 30,154 patients with IPF, the median age category was 75–79 years, and 72.7% patients were male (Table 1 and Additional file: Figure S1). Median survival was 35.2 months (95% confidence interval [CI], 34.5–35.9) (Additional file: Figure S2). During the study period, 14,525 patients (48.2%) were treated with antifibrotics. The 1-year cumulative discontinuation rates for nintedanib and pirfenidone were 35.0% (95% CI, 33.9–36.1) and 35.4% (95% CI, 34.3–36.5), respectively (Additional file: Figure S3). During the study period, 6,431 patients (42.2%) discontinued antifibrotic therapy.

### Models where antifibrotics initiation treated as a time-dependent covariate

The results of multivariate analyses adjusted for baseline variables are presented in Table 2. In the study cohort (Fig. 1A), the intention-to-treat analyses illustrated that nintedanib (adjusted hazard ratio [aHR], 0.85; 95% CI, 0.81–0.89) and pirfenidone (aHR, 0.89; 95% CI, 0.86–0.93) were associated with reduced risk of mortality.

**Table 2:** Multivariable Fine–Gray sub-distribution hazards analysis of mortality in idiopathic pulmonary fibrosis patients who were and were not treated with antifibrotics with time-dependent covariates: study cohort

	No. of patients	HR	95%CI	*p*-value
Unexposed	15,632	Ref	Ref	Ref
Nintedanib	6766			
Unadjusted		0.93	0.89–0.98	0.0027
Adjusted ^a^		0.85	0.81–0.89	< 0.0001
Pirfenidone	7055			
Unadjusted		1.06	1.02–1.10	0.0046
Adjusted ^a^		0.89	0.86–0.93	< 0.0001

The start of antifibrotic treatment was used as a time-dependent covariate

Lung transplantation was treated as a competing event

^a^Adjusted for age, sex, cerebrovascular disease, dementia, acquired immunodeficiency syndrome/human immunodeficiency virus, myocardial infarction, renal disease, congestive heart failure, peripheral vascular disease, chronic pulmonary disease, peptic ulcer, liver disease, diabetes mellitus, hemiplegia or paraplegia, venous thromboembolic disease, pulmonary hypertension, long-term oxygen use, and corticosteroid use at baseline

CI, confidence interval; HR, hazard ratio

### TTE

#### Patient characteristics

The characteristics of antifibrotic-exposed and unexposed patients before adjustment (Fig. 1B) are presented in Table 3. Comparing the findings, the nintedanib- and pirfenidone-exposed patients were younger than unexposed patients. Patients aged ≥ 80 years accounted for 7,009 of 15,632 (44.8%) unexposed, 489 of 2,754 (17.8%) nintedanib-exposed, and 842 of 3,908 (21.5%) pirfenidone-exposed patients (Additional file: Figure S4).

**Table 3:** Comparison of patients with idiopathic pulmonary fibrosis who were treated and not treated with antifibrotics

	Unexposed *N* = 15,632	Nintedanib *N* = 2754	Standardized Difference vs. unexposed	Pirfenidone *N* = 3908	Standardized Difference vs. unexposed
Baseline					
Median age category, years	75–79 ^a^	70–74 ^a^	0.628	70–74 ^a^	0.526
Men	10,980 (70.2)	2053 (74.5)	0.096	2777 (71.1)	0.018
Comorbidity category					
Cerebrovascular disease	4331 (27.7)	592 (21.5)	0.145	830 (21.2)	0.151
Dementia	1358 (8.7)	55 (2.0)	0.301	131 (3.4)	0.226
Acquired immunodeficiency syndrome/ human immunodeficiency virus	< 10	< 10	< 0.05	< 10	< 0.001
Myocardial infarction	1051 (6.7)	132 (4.8)	0.083	205 (5.2)	0.062
Renal disease	1291 (8.3)	127 (4.6)	0.149	220 (5.6)	0.104
Congestive heart failure	5680 (36.3)	823 (29.9)	0.137	1300 (33.3)	0.064
Peripheral vascular disease	3181 (20.3)	481 (17.5)	0.074	723 (18.5)	0.047
Chronic pulmonary disease	10,212 (65.3)	2097 (76.1)	0.239	029 (77.5)	0.272
Peptic ulcer	4889 (31.3)	950 (34.5)	0.069	1391 (35.6)	0.092
Liver disease	3923 (25.1)	803 (29.2)	0.091	1101 (28.2)	0.07
Diabetes mellitus	6943 (44.4)	1415 (51.4)	0.140	1884 (48.2)	0.076
Hemiplegia or paraplegia	271 (1.7)	21 (0.8)	0.088	52 (1.3)	0.033
Charlson comorbidity index			0.066		0.044
0–2	7619 (48.7)	1375 (49.9)		1891 (48.4)	
3–4	5231 (33.5)	975 (35.4)		1374 (35.2)	
≥ 5	2782 (17.8)	404 (14.7)		643 (16.5)	
Venous thromboembolic disease	107 (0.7)	16 (0.6)	0.013	37 (0.9)	0.029
Pulmonary hypertension	453 (2.9)	97 (3.5)	0.035	134 (3.4)	0.03
Long-term oxygen use	1994 (12.8)	535 (19.4)	0.182	810 (20.7)	0.215
Corticosteroid use	3808 (24.4)	677 (24.6)	0.005	1274 (32.6)	0.183
Observation period, months (IQR)	19.1 (6.6–36.7)	20.6 (13.6–31.6)		23.3 (13.3–39.2)	
Antifibrotic therapy					
Nintedanib only	–	2497 (90.7)		–	
Pirfenidone only	–	–		3266 (84.1)	
Switched to another agent	–	257 (9.3)		622 (15.9)	
Therapy duration, months (IQR)	–	13.8 (4.3–23.6)		12.6 (3.1–26.3)	
Discontinuation	–	1204 (43.7)		1826 (46.7)	
Continuation of the same antifibrotic until censoring/death		1372 (49.8)		1656 (42.4)	
Lung transplantation	< 10	< 10		< 10	
Median survival time, months (95% CI)	31.5 (30.2–32.6)	37.5 (35.3–40.6)		32.7 (31.2–34.3)	
Death during the observation period	8005 (51.2)	1079 (39.2)		2088 (53.4)	

Data are presented as categories, number (%), median (interquartile range) or, as median (95% confidence interval)

^a^Details of the distribution of age categories are presented in Additional file: Figure S4

IQR, interquartile range; CI, confidence interval

Compared with the findings in unexposed patients, nintedanib-exposed patients showed lower rates of cerebrovascular disease, dementia, renal disease, and congestive heart failure (standardized differences, 0.145, 0.301, 0.149 and 0.137, respectively) and higher rates of chronic pulmonary disease and diabetes (standardized differences, 0.239 and 0.140, respectively). Pirfenidone-exposed patients showed similar tendency with lower rates of cerebrovascular disease, dementia, and renal disease and congestive heart failure (standardized differences, 0.151, 0.226, and 0.104, respectively) and higher rates of chronic pulmonary disease (standardized difference, 0.239). More than 50% patients in all three groups had a Charlson comorbidity index of ≥ 3, with no significant differences among the groups. Nintedanib- and pirfenidone-exposed patients were more likely to be on long-term oxygen therapy at baseline than unexposed patients (standardized differences, 0.182 and 0.215, respectively). Compared with the findings in unexposed patients, the rate of corticosteroid use at baseline was similar in nintedanib-exposed patients but higher in pirfenidone-exposed patients (standardized differences, 0.005 and 0.183, respectively).

Of the nintedanib-exposed patients, 257 (9.3%) were switched to pirfenidone, and 1,204 (43.7%) eventually discontinued antifibrotic therapy. Of the pirfenidone-exposed patients, 622 (15.9%) were switched to nintedanib, and 1,826 (46.7%) eventually discontinued antifibrotic therapy. The median of survival durations for unexposed, nintedanib-exposed, and pirfenidone-exposed patients were 31.5, 37.5, and 32.7 months, respectively.

#### Association between antifibrotic therapy and mortality: Multivariate Fine–Gray models

The results of multivariate analyses adjusted for baseline variables are presented in Table 4. The intention-to-treat analysis found that nintedanib (aHR, 0.72; 95% CI, 0.67–0.77) and pirfenidone treatments (aHR, 0.86; 95% CI, 0.82–0.91) were associated with reduced risk of mortality.

**Table 4:** Multivariate Fine–Gray sub-distribution hazard analysis of mortality in patients with idiopathic pulmonary fibrosis who were and were not treated with antifibrotics: Target trial emulation

	No. of patients	HR	95%CI	*p*-value
Unexposed	15,632	Ref	Ref	Ref
Nintedanib	2754			
Unadjusted		0.69	0.64–0.73	< 0.0001
Adjusted ^a^		0.72	0.67–0.77	< 0.0001
Pirfenidone	3908			
Unadjusted		0.86	0.82–0.91	< 0.0001
Adjusted ^a^		0.86	0.82–0.91	< 0.0001

Lung transplantation was considered a competing event

^a^Adjusted for age, sex, cerebrovascular disease, dementia, acquired immunodeficiency syndrome/human immunodeficiency virus, myocardial infarction, renal disease, congestive heart failure, peripheral vascular disease, chronic pulmonary disease, peptic ulcer, liver disease, diabetes mellitus, hemiplegia or paraplegia, venous thromboembolic disease, pulmonary hypertension, long-term oxygen use, and corticosteroid use at baseline

CI, confidence interval; HR, hazard ratio

**Fig. 2:**
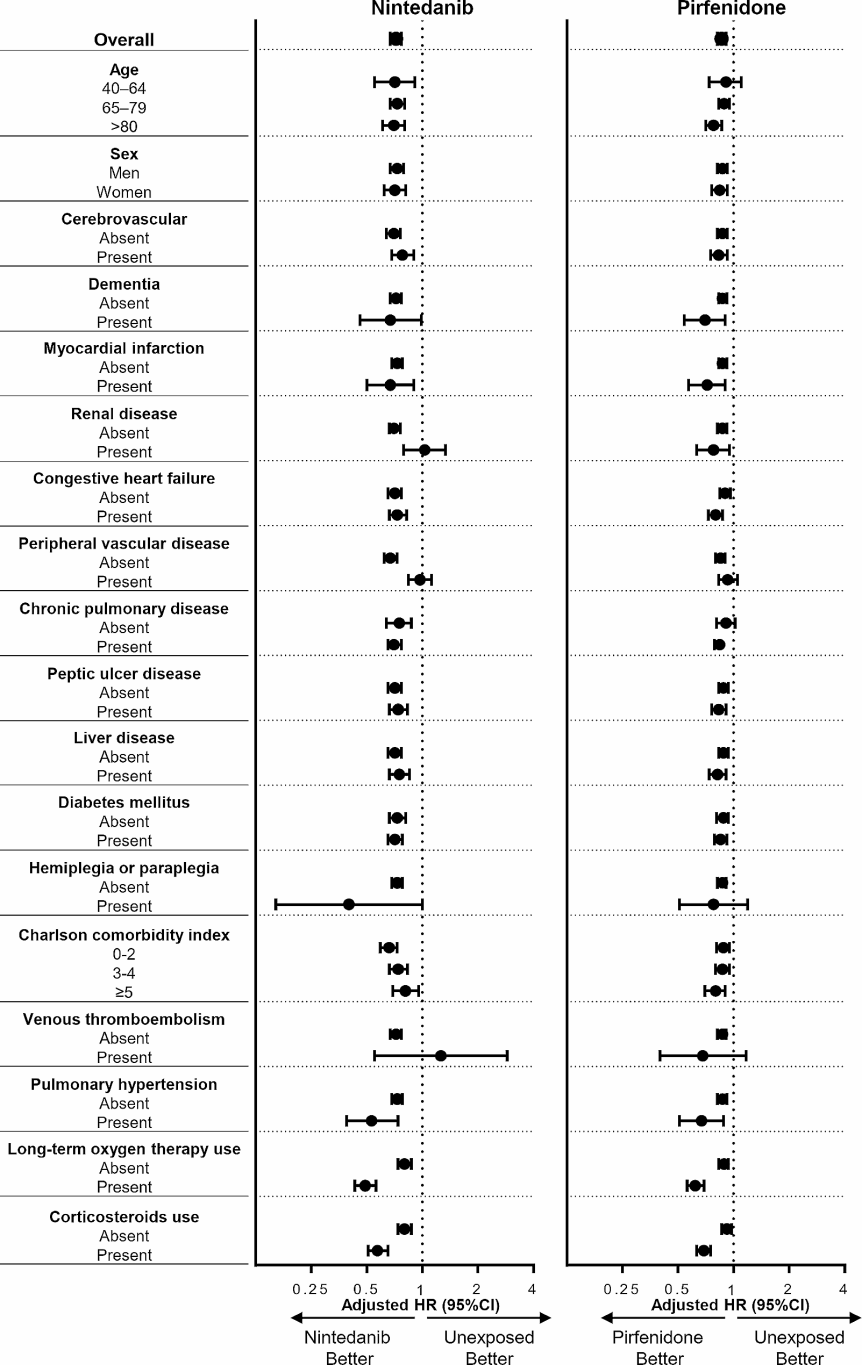
Forest plots of a subgroup intention-to-treat analysis of the effects of nintedanib and pirfenidone on mortality. Full versions of these figures are shown in Additional file: Figures S5 and S6. HR, hazard ratio; CI, confidence interval

Subgroup intention-to-treat analyses of the effects of nintedanib and pirfenidone on mortality are presented using forest plots in Fig. 2 and Additional files: Figures S5 and S6. Nintedanib treatment was associated with reduced risk of mortality regardless of the age categories (40–64, 65–79, and ≥ 80 years), sex, comorbidities, long-term oxygen use, and corticosteroid use at baseline, although this association was not observed in the renal disease and venous thromboembolic disease subgroups. Pirfenidone treatment was consistently associated with a reduced risk of mortality regardless of the factors stated above at baseline.

#### Association between antifibrotic therapy and mortality: propensity score matching comparison

The characteristics of the propensity score-matched antifibrotic-exposed and unexposed groups in the intention-to-treat are presented in Table 5. The characteristics of the antifibrotic-exposed (nintedanib and pirfenidone) and unexposed patients were well matched (with standardized differences for all baseline characteristic variables of < 0.01).

**Table 5:** Propensity score matching for patients with idiopathic pulmonary fibrosis who were or were not treated with antifibrotics

	Unexposed ^a^ *N* = 2523	Nintedanib ^a^ *N* = 2523	Standardized Difference	Unexposed ^a^ *N* = 3399	Pirfenidone ^a^ *N* = 3399	Standardized Difference
Baseline						
Median age category, years	70–74	70–74	< 0.001	70–74	70–74	< 0.001
40–49	20 (0.8)	20 (0.8)		23 (0.7)	23 (0.7)	
50–54	32 (1.3)	32 (1.3)		46 (1.4)	46 (1.4)	
55–59	76 (3.0)	76 (3.0)		104 (3.1)	104 (3.1)	
60–64	175 (6.9)	175 (6.9)		281 (8.3)	281 (8.3)	
65–69	461 (18.3)	461 (18.3)		533 (15.7)	533 (15.7)	
70–74	628 (24.9)	628 (24.9)		766 (22.5)	766 (22.5)	
75–79	679 (26.9)	679 (26.9)		929 (27.3)	929 (27.3)	
80–	452 (17.9)	452 (17.9)		717 (21.1)	717 (21.1)	
Men	1889 (74.9)	1889 (74.9)	< 0.001	2371 (69.8)	2371 (69.8)	< 0.001
Comorbidity category						
Cerebrovascular disease	538 (21.3)	538 (21.3)	< 0.001	710 (20.9)	710 (20.9)	< 0.001
Dementia	50 (2.0)	50 (2.0)	< 0.001	107 (3.1)	107 (3.1)	< 0.001
Myocardial infarction	118 (4.7)	118 (4.7)	< 0.001	174 (5.1)	174 (5.1)	< 0.001
Renal disease	118 (4.7)	118 (4.7)	< 0.001	193 (5.7)	193 (5.7)	< 0.001
Congestive heart failure	741 (29.4)	741 (29.4)	< 0.001	1098 (32.3)	1098 (32.3)	< 0.001
Peripheral vascular disease	442 (17.5)	442 (17.5)	< 0.001	633 (18.6)	633 (18.6)	< 0.001
Chronic pulmonary disease	1913 (75.8)	1913 (75.8)	< 0.001	2617 (77.0)	2617 (77.0)	< 0.001
Peptic ulcer	864 (34.2)	864 (34.2)	< 0.001	1176 (34.6)	1176 (34.6)	< 0.001
Liver disease	734 (29.1)	734 (29.1)	< 0.001	967 (28.4)	967 (28.4)	< 0.001
Diabetes mellitus	1264 (51.3)	1264 (51.3)	< 0.001	1624 (47.8)	1624 (47.8)	< 0.001
Hemiplegia or paraplegia	15 (0.6)	15 (0.6)	< 0.001	44 (1.3)	44 (1.3)	< 0.001
Venous thromboembolic disease	< 10	< 10	< 0.001	32 (0.9)	32 (0.9)	< 0.001
Pulmonary hypertension	81 (3.2)	81 (3.2)	< 0.001	106 (3.1)	106 (3.1)	< 0.001
Long-term oxygen use	433 (17.2)	433 (17.2)	< 0.001	591 (17.4)	591 (17.4)	< 0.001
Corticosteroid use	593 (23.5)	593 (23.5)	< 0.001	1028 (30.2)	1028 (30.2)	< 0.001
Observation period, months (IQR)	19.2 (7.0–36.5)	20.8 (13.9–31.9)		19.2 (7.0–36.5)	24.4 (13.9–40.1)	
Antifibrotic therapy						
Nintedanib only	–	2286 (90.6)		–	–	
Pirfenidone only	–	–			2857 (84.1)	
Switched to another agent	–	237 (9.4)		–	542 (15.9)	
Therapy duration, months (IQR)	–	14.0 (4.4–23.8)		–	12.8 (3.3–26.9)	
Discontinuation	–	1118 (44.3)		–	1630 (48.0)	
Lung transplantation	< 10	< 10		< 10	< 10	
Median survival time, months (95% CI)	34.6 (30.6–39.5)	38.7 (35.8–42.1)		31.6 (29.2–35.0)	34.7 (33.0–36.2)	
Death during the observation period	1226 (48.6)	962 (38.1)		1711 (50.3)	1752 (51.5)	

Data are presented as category, number (%), or as median (interquartile range)

^a^Adjusted for age, sex, cerebrovascular disease, dementia, acquired immunodeficiency syndrome/human immunodeficiency virus, myocardial infarction, renal disease, congestive heart failure, peripheral vascular disease, chronic pulmonary disease, peptic ulcer, liver disease, diabetes mellitus, hemiplegia or paraplegia, venous thromboembolic disease, pulmonary hypertension, long-term oxygen use and corticosteroid use at baseline

The guidelines of the Japanese Ministry of Health, Labour and Welfare prohibit the publication of specific numbers for variables with fewer than 10 patients for anonymity reasons. Therefore, variables with fewer than 10 patients were denoted as < 10

IQR, interquartile range; CI, confidence interval

**Fig. 3:**
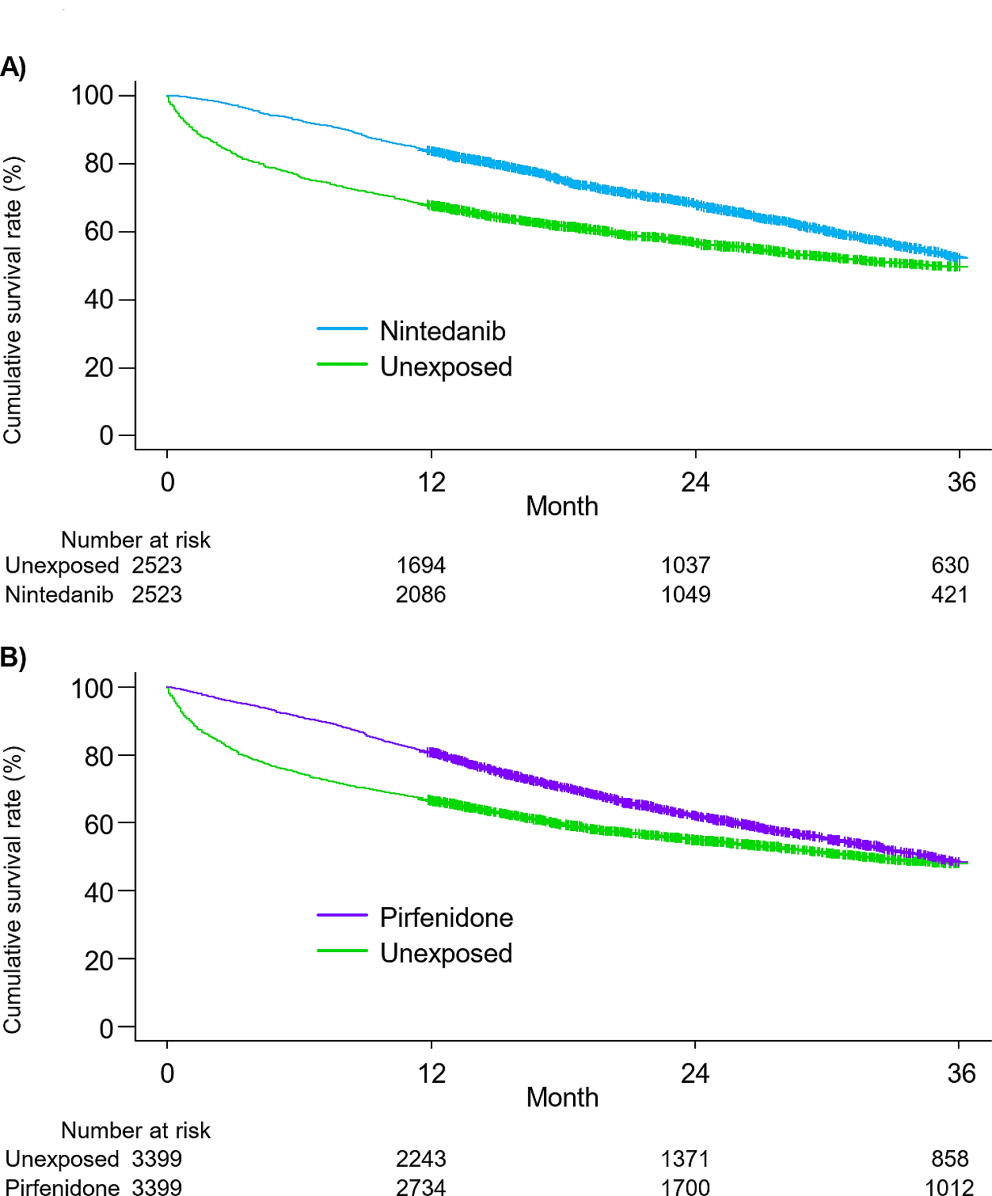
Intention-to-treat survival curves of propensity score-matched idiopathic pulmonary fibrosis patients who were or were not treated with antifibrotics. (**A**) The nintedanib-exposed group had a significantly higher survival rate than the unexposed group (HR, 0.74; 95% CI, 0.68–0.80; *p* < 0.0001). The median survival times of the nintedanib-exposed and unexposed groups were 38.7 and 34.6 months, respectively. (**B**) The pirfenidone-exposed group had a significantly higher survival rate than the unexposed group (HR, 0.86; 95% CI, 0.81–0.92; *p* < 0.0001). The median survival times of the pirfenidone-exposed and unexposed groups were 34.7 and 31.6 months, respectively. HR, hazard ratio; CI, confidence interval

Figure 3A and B presents the survival curves for the intention-to-treat analyses of antifibrotic therapy.

The propensity score-matched nintedanib-exposed group exhibited longer median survival than the propensity score-matched unexposed group (38.7 and 34.6 months, respectively; *p* < 0.01), and nintedanib treatment was associated with reduced mortality (HR, 0.74; 95% CI, 0.68–0.80). The propensity score-matched pirfenidone-exposed group showed longer median survival than the propensity score-matched unexposed group (34.7 and 31.6 months, respectively; *p* < 0.01), and pirfenidone treatment was associated with reduced mortality (HR, 0.86; 95% CI, 0.81–0.92).

## Discussion

This study is the first and largest to demonstrate the association of antifibrotic therapy (nintedanib and pirfenidone) with long-term mortality in patients with IPF in the real-world using methods that control for immortal time bias. Additionally, subgroup analyses were conducted to provide further insights. One strength of this study is the robustness of its results, demonstrated using multiple approaches that control for immortal time bias. Another strength of this study is that it included almost all patients diagnosed with IPF in Japan, i.e., not only the typical patients accessible to hospitals specializing in ILD and eligible under strict criteria, such as those enrolled in clinical studies, but also elderly/very elderly patients, those with multiple comorbidities, and those who were not accessible or referred to hospitals specializing in ILD. Therefore, it represents a diverse and broad population, making it more representative of patients with IPF in the real world. Furthermore, we have demonstrated for the first time that nintedanib is associated with a reduction in long-term mortality rates on an intention-to-treat basis.

Intention-to-treat analyses are considered more representative of treatment effects. Although most IPF patients remain with their first-line antifibrotic treatment, some are switched to a different antifibrotic agent, whereas others discontinue treatment because of adverse effects. Several observational studies using intention-to-treat analyses have reported a reduced risk of mortality from antifibrotic treatment in general [15–26] or from pirfenidone specifically [27, 28]. However, as noted in the Introduction, none of these studies controlled for immortal time bias, resulting in a need for reanalysis that adequately addresses this bias [30–32]. Additionally, although a pooled analysis predicted the long-term effects of nintedanib treatment based on short-term mortality data, [9] there have been no previous studies of its long-term effectiveness. The current study, which controlled for immortal time bias, has demonstrated that antifibrotic therapy is associated with a reduced risk of long-term mortality. Notably, the results of our intention-to-treat analysis demonstrated that first-line treatment with either nintedanib or pirfenidone provides similar mortality reductions. Although all-cause mortality is the most clinically meaningful endpoint for both patients and clinicians, the relatively rare, progressive, and fatal nature of IPF makes it extremely difficult to conduct RCTs that compare the mortality-reducing effects of drugs with placebos [38–40]. Therefore, our robust results showing antifibrotic treatment of IPF to improve survival rather than prevent respiratory function deterioration are of particular value.

Previous research using RCTs to determine the effects of antifibrotics [2–7] has implemented strict criteria that have excluded patients aged > 75–80 years and those with comorbidities. Observational studies that have reported reductions in mortality rates owing to antifibrotic treatment have included elderly patients and those with comorbidities but have failed to conduct separate analyses of each subgroup. Importantly, for patients with IPF, the presence of certain comorbidities has reportedly been associated with mortality regardless of age, gender, and respiratory function, but most of those observational studies did not consider comorbidity as an adjustment factor in their analyses of the mortality-reducing effects of antifibrotics. However, this study addressed these issues, by including older adults and those with comorbidities in the cohort, by performing multivariate analyses adjusted for age, gender, and comorbidity categories, and by conducting subgroup analyses to identify differences in the relationship between antifibrotic therapy and mortality between these subgroups. Interactions between antifibrotic treatment outcomes and clinical characteristics of patients were observed in several subgroups (e.g., renal disease and peripheral vascular disease for nintedanib), suggesting that the mortality-reducing effects of the corresponding antifibrotics are attenuated in these subgroups. However, despite such interactions, with some exceptions, nintedanib and pirfenidone therapies were consistently associated with reduced mortality risks, even in patients over 80, regardless of the type of comorbidity, the presence of multiple comorbidities, or treatment with long-term oxygen therapy or corticosteroids. These results demonstrate that antifibrotic therapy reduces the risk of mortality even in a broad IPF population that is not limited by the strict criteria employed in previous RCTs.

This study investigated the association between antifibrotic therapy and mortality in patients with IPF. However, leveraging the extensive data accumulated in the NDB may yield additional valuable insights. For example, determining whether pirfenidone or nintedanib demonstrates better efficacy in reducing mortality or identifying preferable antifibrotic agent based on subgroups could provide useful information for the selection of antifibrotic treatments. Moreover, shifting focus to ILDs other than IPF, nintedanib has recently been proven effective in preventing pulmonary function deterioration in patients with progressive fibrosing interstitial lung disease (PF-ILD)/progressive pulmonary fibrosis (PPF) [1, 41, 42]. Investigating whether nintedanib reduces mortality rates in patients with PF-ILD/PPF using this study’s approach could yield intriguing results. Thus, further investigation on this subject is necessary.

This study had several limitations. First, this was a retrospective study. Therefore, the treatment strategy of whether to initiate antifibrotic therapy at the time of IPF diagnosis has not been randomized. However, antifibrotic therapy for patients with IPF were not recommended in the 2011 guideline and were only conditionally recommended in the 2015 guideline. Note that the study period, 2013–2018, was a time when antifibrotics were not used as aggressively in patients for whom they would have been recommended today. It is presumed that among the patients who did not receive antifibrotic therapy there were not only those who were ineligible for antifibrotic therapy due to severe comorbidities, but also those who did not initiate the treatment due to mild disease; and conversely, among those who did receive antifibrotic therapy, some patients initiated the treatment due to advanced stage of the disease. Therefore, this study attempted to minimize this limitation by adjusting for baseline confounders using the TTE framework and multivariate analysis. Second, the results of this study were based on claims data. The IPF diagnoses were made by the attending physician at each hospital, but it could not be established whether these diagnoses were based on multidisciplinary discussion (MDD). However, in the real world, MDD is not necessarily possible in all hospitals. In this study, we attempted to increase the specificity of IPF diagnosis by selecting patients using an ICD-10 code for IPF who did not have a baseline or prior history of diseases that can cause secondary ILD, as described in the Additional file: Appendix S1. We believe that this study is of particular significance as it provides external validation about the survival benefits of antifibrotic therapy and their applicability to the patients diagnosed by MDD teams as well as populations that reflect the real-world clinical practice. Third, the NDB does not contain information on the results of clinical/physiological tests. Therefore, the results of pulmonary function tests, such as the forced vital capacity, were unavailable in this study. To minimize this limitation, the presence or absence of long-term oxygen therapy was used as an adjustment factor instead of pulmonary function test results in our multivariate analysis of mortality. Additionally, this study included baseline comorbidities as adjustment factors. Recently, it has been noted that the presence of comorbidities has a significant impact on survival of patients with IPF [35, 43]. However, the observed survival benefits of antifibrotic therapy as reported by previous observational studies did not involve adequate adjustment for comorbidities and immortal time bias. Therefore, this study has an advantage over previous studies in this respect. Fourth, we did not analyze antifibrotic dose reductions in each patient or the effects of dose reductions on mortality. Additionally, some patients switched from nintedanib to pirfenidone and vice-versa; however, the database does not contain information on the reasons for these switches. Therefore, we could not evaluate the impact of these switches on mortality. Finally, information about the cause of death was not available. Therefore, the impact of antifibrotic treatment on causes of death could not be analyzed.

In conclusion, this large-scale real-world study is the first to externally validate that antifibrotic treatment is associated with reduced risk of long-term mortality in patients with IPF regardless of age, sex, comorbidity, long-term oxygen use, or corticosteroid use, excluding some subgroups, using multiple methods to avoid immortal time bias. The robust results of this study might support the use of antifibrotic therapy for various IPF populations in terms of improved survival. We hope that our findings will help clinicians, patients, and their families and aid effective treatment decisions.

### Electronic supplementary material

Below is the link to the electronic supplementary material.

Supplementary Material 1

## Data Availability

No datasets were generated or analysed during the current study.

## Declarations

### Generative AI and AI-assisted technologies in the writing process

None of Generative AI and AI-assisted technologies were used in this manuscript.
