# Supplementary material for: Antifibrotics and mortality in idiopathic pulmonary fibrosis: external validity and avoidance of immortal time bias
Source: Respir Res. 2024 Jul 31;25:293. doi: 10.1186/s12931-024-02922-y (PMC11293013; doi:10.1186/s12931-024-02922-y)
Supplement: Supplementary file 1 — Supplementary Material 1 [file 12931_2024_2922_MOESM1_ESM.docx]

**Additional files**

- **Appendix S1**
- **Appendix S2**
- **Appendix S3**
- **Table** S**1. List of ICD-10 codes for comorbidities used to calculate the Charlson Comorbidity Index**
- **Table S2. Specification and emulation of targeted trial in the study of antifibrotic treatment and mortality in idiopathic pulmonary fibrosis patients**
- **Figure S1. Distribution of age categories in a study cohort of patients with idiopathic pulmonary fibrosis**
- **Figure S2. Kaplan–Meier survival curve for a study cohort of patients with idiopathic pulmonary fibrosis**
- **Figure S3.** **The cumulative discontinuation rate of antifibrotic therapy in patients with idiopathic pulmonary fibrosis**
- **Figure S4. Distribution of age categories in patients with idiopathic pulmonary fibrosis who were or were not treated with antifibrotics**
- **Figure S5. Forest plots of a subgroup intention-to-treat analysis of patients with idiopathic pulmonary fibrosis treated with nintedanib**
- **Figure S6. Forest plots of a subgroup intention-to-treat analysis of patients with idiopathic pulmonary fibrosis treated with pirfenidone**
- **References**

**Appendix S1**

The Japanese National Claims Database includes demographic and clinical patient information, including the International Statistical Classification of Diseases and Related Health Problems–Tenth Revision (ICD-10) codes for patient diagnoses, prescribed drugs, and information about patient mortalities. From the patients registered on the database, we identified 41,891 diagnosed with idiopathic pulmonary fibrosis (IPF) between 2013 and 2018 using the following algorithm, which is a slight modification of the algorithm of Raghu et al.,^1^ to increase the specificity of patients with IPF: 1) presence of an ICD-10 code for IPF (J84.112); 2) ≥40 years old; 3) and absence of an ICD-10 code for secondary interstitial lung disease (ILD) or diseases that can cause secondary ILD, such as collagen disease, vasculitis, pneumoconiosis, and hypersensitivity pneumonitis (C96.6, D219, J84.112, and J60–709, with the exceptions of J840, M05–06, M32–34, M313, M315, M317–318, M350–351, M353, and M360) (entire cohort). Of these, we excluded 11,737 patients with ICD-10 codes for malignant or metastatic tumors (C00–26, C30–34, C37–41, C43, C45–58, C60–76, C77–80, C81–85, C88, C90–97) at the time of IPF diagnosis Figure **1**A, Table S1). Consequently, 30,154 patients with IPF were enrolled (study cohort). For all enrolled patients, we extracted data on any comorbid diseases used to calculate the Charlson Comorbidity Index (CCI), which is a commonly used risk-adjustment variable in studies investigating treatment outcomes (Table S1).^2^ One point was assigned to each comorbidity category, and each patient’s total CCI was calculated. We further extracted data on venous thromboembolic disease (ICD-10 codes: I269, I802) and pulmonary hypertension (ICD-10 codes: I270 and I272), which have been reported as prognostic factors for IPF.^3, 4^ Patients were censored if they remained alive until December 31, 2019.

**Appendix S2**

**Avoidance of immortal time bias**

Treatment records for patients with IPF were extracted from the databases and information on their treatment with nintedanib and pirfenidone was recorded, including the dates of initiation and discontinuation of each drug.

The methods used in this study to avoid immortal time bias included 1) models with drug initiation treated as a time-dependent covariate^5^ and 2) target trial emulation (TTE) framework,^6^ both having new user designs for antifibrotics.

The first model employed Fine–Gray models with antifibrotic initiation as a time-dependent covariate to investigate the effects of antifibrotics on the study cohort. The intention-to-treat analysis included all patients who did not use antifibrotics between the date of IPF diagnosis (i.e., baseline) and the date of censoring or death (unexposed patients) and all patients assigned to first-line antifibrotic treatment with nintedanib or pirfenidone between the date of IPF diagnosis and the date of censoring or death.

The second model was a TTE framework that emulated the target trial. First, a hypothetical but pragmatic clinical trial was designed to answer the clinical question of interest (target trial specification); subsequently a TTE framework was designed to approximate the target trial using the available observational data. Patients meeting the eligibility criteria specified in the TTE framework were then enrolled and analyzed. This study emulated a trial comparing mortality between antifibrotic-exposed and unexposed patients (i.e., a targeted trial). To avoid immortal time bias, we designated the date when patients met the eligibility criteria as time zero. Patients whose allocation to the treatment strategy coincided with this time zero date were identified, and follow-up was initiated on the same date (i.e., time zero; Table S2). Notably, this model included “unexposed patients” and patients who initiated antifibrotic treatment on the date of IPF diagnosis (nintedanib- or pirfenidone-exposed patients; Figure 1B). The intention-to-treat analysis included the unexposed patients and all patients assigned to first-line antifibrotic treatment (nintedanib or pirfenidone). Multivariate Fine–Gray models were used in the intention-to-treat analyses. The results were validated by propensity score matching (Figure 1C). More information on the adjustment for confounders is provided in the section titled Supplementary Statistical Analysis.

**Appendix S3**

Continuous and categorical variables were expressed as median (interquartile range [IQR]) and number (%), respectively. Age data were expressed as ranges (in increments of 5 years) in accordance with the Japanese Ministry of Health, Labour and Welfare guidelines for the use of claims data. Chi-squared tests were performed for between-group comparisons of proportions. The observation period was calculated from the date of IPF diagnosis to censoring or death. Survival rates were calculated using Gray’s method, in which lung transplantation is treated as a competing event. The cumulative antifibrotic discontinuation rate was determined using Gray’s method, in which death is treated as a competing event. Multivariate Fine–Gray proportional hazards regression models (with/without antifibrotic initiation treated as a time-dependent covariate) were used to determine the association between antifibrotic treatment and mortality, in which lung transplantation is treated as a competing event. After adjustments for age, gender, cerebrovascular disease, dementia, acquired immunodeficiency syndrome/human immunodeficiency virus, myocardial infarction, renal disease, congestive heart failure, peripheral vascular disease, chronic pulmonary disease, peptic ulcer, liver disease, diabetes mellitus, hemiplegia or paraplegia, venous thromboembolic disease, pulmonary hypertension, long-term oxygen use and corticosteroid use, the hazard ratios (HR), 95% confidence intervals (CI), and *p*-values were calculated. The subgroup analysis results were presented as forest plots. A propensity score-matched comparison was also performed between those treated with antifibrotics and those who were not. Propensity scores were calculated using a logistic regression model adjusted for age, gender, cerebrovascular disease, dementia, acquired immunodeficiency syndrome/human immunodeficiency virus, myocardial infarction, renal disease, congestive heart failure, peripheral vascular disease, chronic pulmonary disease, peptic ulcer, liver disease, diabetes mellitus, hemiplegia or paraplegia, venous thromboembolic disease, pulmonary hypertension, long-term oxygen use, and corticosteroid use. Matching was performed using the following algorithm: 1:1 nearest neighbor match with a caliper width of 0.2 of the standard deviation of the logit of the propensity score with no replacement. In all analyses, *p*<0.05 was considered statistically significant. Because of the large sample size, standardized differences were used to assess differences in baseline variables between the two groups. When the standardized difference was <0.1, the variables of the two groups were considered approximately equivalent, even if the *p*-value was significant. All data were analyzed using JMP, version 13.2.1 (SAS Institute Inc., Cary, NC, USA); R software, version 4.2.1 (The R Foundation for Statistical Computing, Austria); and Prism, version 7.04 (GraphPad Software Inc., San Diego, CA, USA).

**Table** S**1.** **List of ICD-10 codes for comorbidities used to calculate the Charlson Comorbidity Index**

| **Comorbidity** | **ICD-10 code** |
| --- | --- |
| Cerebrovascular disease | G45–46, I60–69, H340 |
| Any malignancy ^a^ | C00–26, C30–34, C37–41, C43, C45–58, C60–76, C81–85, C88, C90–97 |
| Dementia | F00–03, F51, G30, G311 |
| AIDS/ HIV | B20–22, B24 |
| Myocardial infarction | I21–22, I252 |
| Renal disease | N18–19, I120, I131, N032–N037, N052–N057, N250, Z490–492, Z940, Z992 |
| Congestive heart failure | I43, I50, I099, I110, I130, I132, I255, I420, I425–429, P290 |
| Peripheral vascular disease | I70–71, I731, I738–739, I771, I790, I792, K551, K558–559, Z958–959 |
| Chronic pulmonary disease | J40–47, J60–67, I278–279, J684, J701, J703 |
| Rheumatic disease | M05–06, M32–34, M315, M 351, M353, M360 |
| Peptic ulcer | K25–28 |
| Liver disease | B18, I850, I859, I864, I982, K700–704, K709, K711, K713–715, K717, K721, K729, K760, K762–769, Z944 |
| Diabetes mellitus | E10–14 |
| Hemiplegia or paraplegia | G81–82, G041, G144, G801–802, G830–834, G839 |
| Metastatic solid tumours | C77–80 |

^a^All malignancies, including lymphoma and leukemia, but excluding malignant neoplasms of the skin.

AIDS, acquired immunodeficiency syndrome; HIV, human immunodeficiency virus; ICD-10, International Classification of Diseases–Tenth Revision

**Table S2. Specification and emulation of targeted trial in the study of antifibrotic treatment and mortality in idiopathic pulmonary fibrosis patients**

| **Protocol** | **Target trial specification** | **Target trial emulation** |
| --- | --- | --- |
| Eligibility criteria | - Patients diagnosed with IPF - No malignant or metastatic tumours at baseline - No history of antifibrotic therapy   Baseline (i.e., time zero) defined as the date on which all eligibility criteria were met | - Patients with an IPF ICD-10 code (see Supplementary Materials) - No malignant or metastatic tumours at baseline - No history of antifibrotic therapy   Baseline (i.e., time zero) defined as the date on which all eligibility criteria were met |
| Treatment strategies | 1. Antifibrotic therapy (nintedanib or pirfenidone) initiated at baseline and continued until an event occurs that prevents therapy continuation 2. No antifibrotics initiated during the follow-up period, beginning at baseline | Same as for the target trial  The antifibrotic start date defined as the first date of prescription |
| Treatment assignation | Individuals will be randomly assigned to a treatment group at baseline. Individuals and their treating physicians will be aware of the assigned treatment strategy | Individuals will be nonrandomly assigned to a treatment group at baseline.  Randomisation is emulated by adjusting for confounders |
| Outcomes | All-cause death | Same as for the target trial |
| Follow-up | Start at baseline (i.e., time zero) and end at death or censoring, whichever occurs first | Same as for the target trial |
| Causal contrasts | Intention-to-treat effects | Observational analogue of intention-to-treat effects |
| Analytic plan | - Fine–Gray proportional hazards regression model - Comparison of mortality using Kaplan–Meier curves | - Fine–Gray proportional hazards regression model adjusted for confounders - Comparison of mortality using Kaplan–Meier curves after propensity score matching |

ICD-10, International Classification of Diseases–Tenth Revision; IPF, idiopathic pulmonary fibrosis

**Figure** **S1. Distribution of age categories in a study cohort of patients with idiopathic pulmonary fibrosis**

Of the 30,154 patients with idiopathic pulmonary fibrosis, 89 (0.3%), 146 (0.5%), 352 (1.2%), 758 (2.5%), 1958 (6.5%), 4142 (13.7%), 5882 (19.5%), 7110 (23.6%), and 9717 (32.2%) were 40–44, 45–49, 50–54, 55–59, 60–64, 65–69, 70–74, 75–79, and ≥80 years old, respectively.

**Figure S2. Kaplan–Meier survival curve for a study cohort of patients with idiopathic pulmonary fibrosis**

The median survival duration was 35.2 (95% confidence interval, 34.5–35.9) months

**Figure S3.** **The cumulative discontinuation rate of antifibrotic therapy in patients with idiopathic pulmonary fibrosis**

The 1-year cumulative discontinuation rates of nintedanib and pirfenidone therapies were 35.0% (95% confidence interval, 33.9%–36.1%) and 35.4% (95% confidence interval, 34.3%–36.5%), respectively.

**Figure S4. Distribution of age categories in patients with idiopathic pulmonary fibrosis who were or were not treated with antifibrotics**

Of the 15,632 unexposed patients, 117 (0.7%), 148 (1.0%), 264 (1.7%), 742 (4.8%), 1560 (10.0%), 2376 (15.2%), 3416 (21.9%), and 7009 (44.8%) were 40–49, 50–54, 55–59, 60–64, 65–69, 70–74, 75–79, and ≥80 years old, respectively.

Of the 2754 patients who received first-line treatment with nintedanib, 24 (0.9%), 41 (1.5%), 87 (3.2%), 195 (7.1%), 502 (18.2%), 684 (24.8%), 732 (26.6%), and 489 (17.8%) were 40–49, 50–54, 55–59, 60–64, 65–69, 70–74, 75–79, and ≥80 years old, respectively.

Of the 3908 patients who received first-line treatment with pirfenidone, 28 (0.7%), 56 (1.4%), 121 (3.1%), 331 (8.5%), 610 (15.6%), 875 (22.4%), 1045 (26.7%), and 842 (21.5%) were 40–49, 50–54, 55–59, 60–64, 65–69, 70–74, 75–79, and ≥80 years old, respectively.

**Figure S5. Forest plots of a subgroup intention-to-treat analysis of patients with idiopathic pulmonary fibrosis treated with nintedanib**


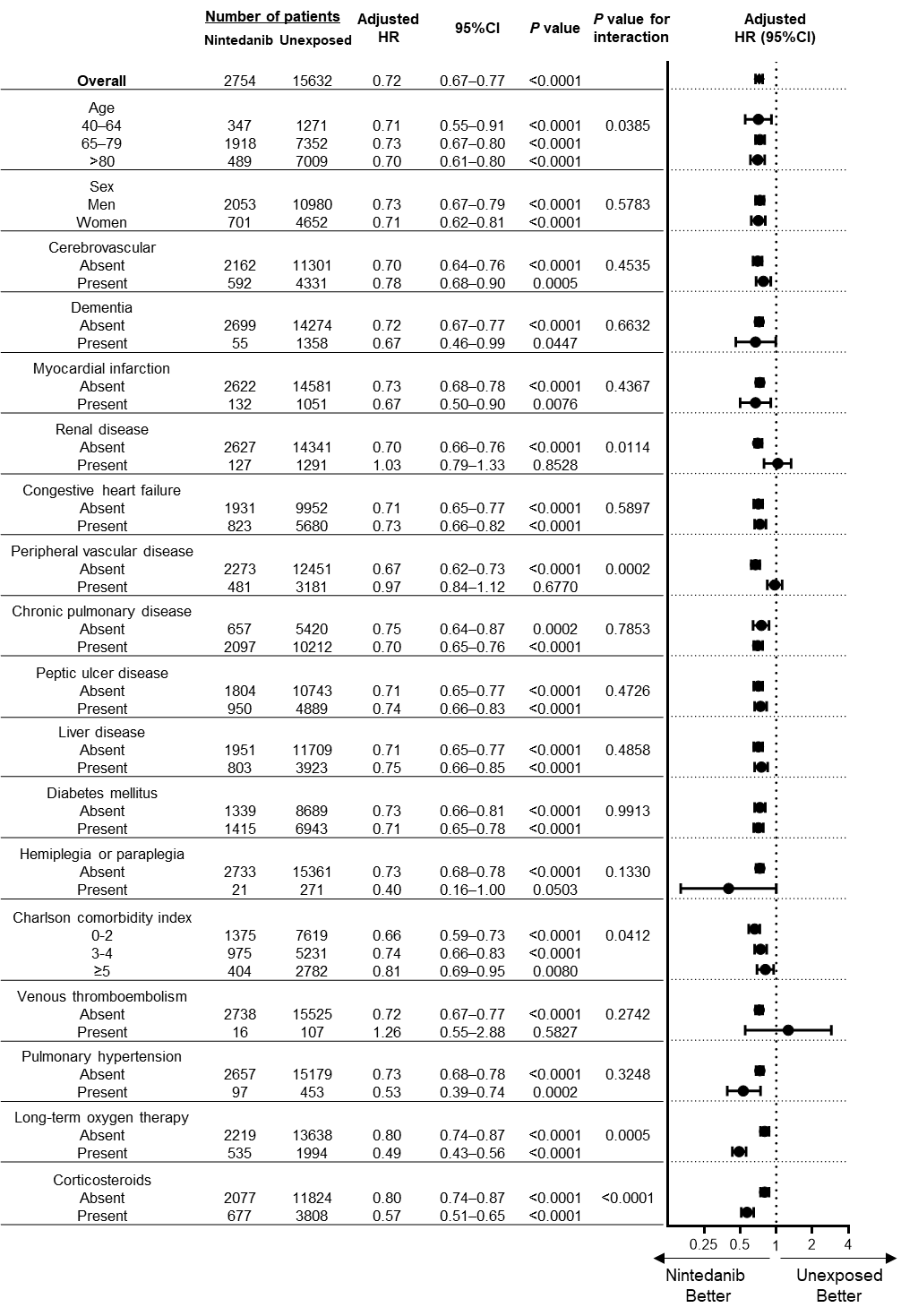


**Figure S6. Forest plots of a subgroup intention-to-treat analysis of patients with idiopathic pulmonary fibrosis treated with pirfenidone**


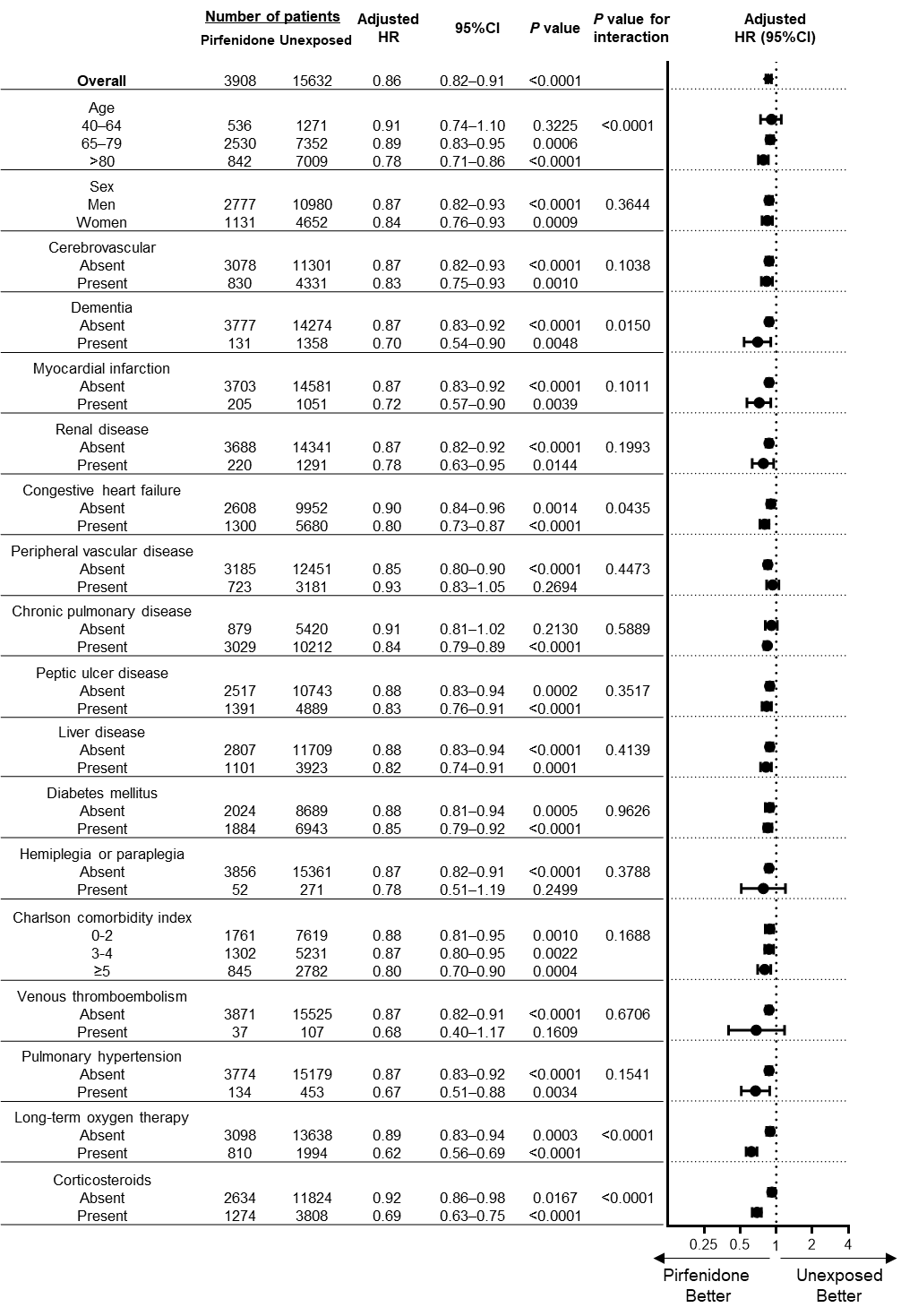


**References**

1 Raghu G, Chen SY, Hou Q, Yeh WS, Collard HR. Incidence and prevalence of idiopathic pulmonary fibrosis in US adults 18-64 years old. The European respiratory journal. 2016; **48**: 179-86.

2 Quan H, Sundararajan V, Halfon P, Fong A, Burnand B, Luthi JC, Saunders LD, Beck CA, Feasby TE, Ghali WA. Coding algorithms for defining comorbidities in ICD-9-CM and ICD-10 administrative data. Medical care. 2005; **43**: 1130-9.

3 King CS, Nathan SD. Idiopathic pulmonary fibrosis: effects and optimal management of comorbidities. The Lancet Respiratory medicine. 2017; **5**: 72-84.

4 Oldham JM, Collard HR. Comorbid Conditions in Idiopathic Pulmonary Fibrosis: Recognition and Management. Frontiers in medicine. 2017; **4**: 123.

5 Suissa S, Suissa K. Antifibrotics and Reduced Mortality in Idiopathic Pulmonary Fibrosis: Immortal Time Bias. American journal of respiratory and critical care medicine. 2022.

6 Hernán MA, Sauer BC, Hernández-Díaz S, Platt R, Shrier I. Specifying a target trial prevents immortal time bias and other self-inflicted injuries in observational analyses. Journal of clinical epidemiology. 2016; **79**: 70-5.
